# Effects of patient selection on the applicability of results from a randomised clinical trial (EORTC 10853) investigating breast-conserving therapy for DCIS

**DOI:** 10.1038/sj.bjc.6600514

**Published:** 2002-09-09

**Authors:** N Bijker, J L Peterse, I S Fentiman, J-P Julien, A A M Hart, A Avril, L Cataliotti, E J T Rutgers

**Affiliations:** Department of Radiation Oncology, The Netherlands Cancer Institute Plesmanlaan 121, 1066 CX Amsterdam, The Netherlands; Department of Pathology, The Netherlands Cancer Institute Plesmanlaan 121, 1066 CX Amsterdam, The Netherlands; Department of Surgery, The Netherlands Cancer Institute, Plesmanlaan 121, 1066 CX Amsterdam, The Netherlands; Guy's Hospital, ICRF Clin. Oncology Unit, St. Thomas Street, London SE1 9RT, UK; Centre Henri Becquerel, Department of Surgery, 1, Rue d'Amiens, 76038 Rouen, France; Department of Surgery, Institut Bergonie, 180, rue de Saint-Genes, 33076 Bordeaux, France; Policlinico di Careggi, Viale Morgagni, 85, 50134 Firenze, Italy

**Keywords:** ductal carcinoma in situ (DCIS), breast-conserving therapy, randomised clinical trial, patient selection

## Abstract

Selection of patients for randomised clinical trials may have a large impact on the applicability of the study results to the general population presenting the same disorder. However, clinical characteristics and outcome data on non-entered patients are usually not available. The effects of patient selection for the EORTC 10853 trial investigating the role of radiotherapy in breast conserving therapy for ductal carcinoma *in situ* have been studied, in an analysis of all patients treated for ductal carcinoma *in situ* in five participating institutes. The reasons for not entering patients were evaluated and treatment results of the randomised patients were compared to those not entered. A total of 910 patients were treated for ductal carcinoma *in situ*. Of these, 477 (52%) were ineligible, with the size of the lesion being the main reason for ineligibility (30% of all ductal carcinoma *in situ*). Of the 433 eligible patients, 278 (64%) were randomised into the trial. The main reasons for non-entry of eligible patients were either physicians' preference for one of the treatment arms (26%) or patients' refusal (9%). These percentages showed significant variation among the institutes. At 4 years follow-up, those patients not entered in the trial and treated with local excision and radiotherapy, had higher local recurrence rates than the patients randomised in the trial and treated with the same approach, (17 *vs* 2%, *P*=0.03). The patients treated with local excision alone had equal local recurrence rates (11% in both groups). Selection of patients may explain the differences in outcome of the randomised patients, and those not-entered. Thus, the results of this trial may not be applicable to all patients with ductal carcinoma *in situ*.

*British Journal of Cancer* (2002) **21**, 615–620. doi:10.1038/sj.bjc.6600514 www.bjcancer.com

© 2002 Cancer Research UK

## 

Results of randomised clinical trials usually form the main source for defining evidence based treatment. Because it is necessary to define clearly the criteria for eligibility, patient selection may produce a trial population which differs substantially from the general population with the disease to whom the results of the trial are intended to be applied. Not only trial eligibility criteria may determine the study population, but also factors such as patient's and doctor's preferences may have an impact. For these reasons, a comparison of patients entered in a trial with all the non-entered cases with the disease for which the trial was designed may give insight in the selection process and therefore in the interpretation and general applicability of the trial results (Anonymous, 1994). This information is hardly ever available, as trials usually do not include registration and follow-up of non-entered patients with the disease.

Between 1986 to 1996, the European Organization for Research and Treatment of Cancer (EORTC) accrued patients in a phase III randomised clinical trial (RCT) to investigate the role of radiotherapy in breast conserving treatment (BCT) of ductal carcinoma *in situ* (DCIS) of the breast. The first results, indicating that radiotherapy reduced the rate of both non-invasive and invasive local recurrence, have been published recently (Julien *et al*, 2000).

There were large differences between the participating institutes in the number of randomised patients: some centres entered over 100 patients in 6 years of participation, whereas others included only a few patients during the whole 10 year period of the trial. These differences could not be explained by the eligibility criteria of the trial. This raised the question as to whether there were differences in the rate of patients diagnosed with DCIS, and whether additional criteria were used to select patients for the trial. This might influence the applicability of the trial results to all patients with DCIS.

The criteria for selection were studied in five participating institutes: Centre Henri Becquerel in Rouen, Policlinico di Careggi in Florence, Guy's Hospital in London, Institut Bergonié in Bordeaux and The Netherlands Cancer Institute in Amsterdam. The objectives of this study were to analyse entry rates and the reasons for non-entry in these centres, to compare treatment results of entered and non-entered patients, and to study possible effects on the applicability of the trial results.

## PATIENTS AND METHODS

A detailed description of the EORTC DCIS trial (protocol 10853) has been given previously (Julien *et al*, 2000). In summary, patients with clinically or mammographically detected DCIS with a maximum diameter of 5 cm were, following histologically confirmed complete local excision of the lesion, randomised between either no further treatment or radiotherapy, 50 Gray in 25 fractions to the whole breast. Exclusion criteria were age over 70 years, a prior or concomitant malignancy other than basal cell carcinoma of the skin or cone biopsied carcinoma *in situ* of the cervix, WHO performance status ⩾2 or mental conditions or social circumstances precluding long-term follow-up, and patients with Paget's disease of the nipple.

Between 1986 and 1996 1010 patients were randomised by 46 institutes from 13 different countries.

The medical records were reviewed of all patients who were treated for DCIS of the breast in five institutes during the period of trial participation. These institutes were responsible for the entry of 27% of all patients in the trial. The five centres were selected because they each have a complete prospective cancer registration system, enabling the identification of all patients treated for DCIS. The number of DCIS cases treated per year in each institute during the period of trial participation was divided by the total number of operable breast cancer cases, to calculate the incidence of DCIS. Entry rates were calculated by dividing the number of randomised patients by the total number of patients treated for DCIS in each institute. Reasons for non-entry, as well as data on method of detection, treatment, follow-up and outcome were obtained by review of the medical records.

Apart from reasons for ineligibility, also doctor's and patient's preferences for trial participation or a particular treatment were searched for. In eligible, but not entered patients, comments on the reason for non-entry were searched in the medical file. Apart from patient's refusal, which was always well documented, it could be stated that: ‘this type of DCIS requires additional radiotherapy', or ‘this subtype is very indolent, and therefore radiotherapy seems to be overtreatment’, in which case this was considered indicative for the reason for non-entry.

Reasons for non-entry were compared between the five institutes by cross tabulation and *P*-values were calculated by the χ^2^ test. Local recurrence-free intervals were defined as the time between the date of randomisation (for the randomised patients) or the date of primary treatment (for the non-entered patients) and the date of recurrence of disease in the ipsilateral breast. Local recurrence-free interval rates were calculated using the Kaplan Meier technique (Kaplan and Meier, 1958), and compared using a 2-sided log-rank test. The institutes are randomly referred to as A, B, C, D and E.

## RESULTS

The average number of patients with operable breast cancer treated per year in the period of participation to the trial varied from 150 to 600 between the five institutes. The incidence of DCIS varied from 5% in institute B and C, to 7% (A and E) to 10% (institute D). In the study period 910 patients with DCIS were treated in the five centres. Of these, 477 (52%) were not entered based on eligibility criteria. The rate of ineligible patients varied between the institutes from 44 to 66% (Table 1

**Table 1:**
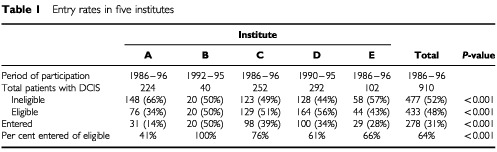
Entry rates in five institutes

). Of the 433 eligible patients 278 (64%) were actually randomised, with large differences between the institutes, varying from 41 to 100%. Ultimately, 278 of 910 patients (31%) entered the trial; the entry rates (number of randomised *vs* total number of patients with DCIS) varied from 14 to 50%.

Table 2a

**Table 2a:**
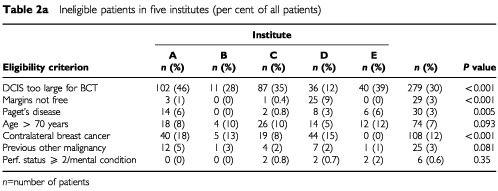
Ineligible patients in five institutes (per cent of all patients)

shows the reasons for non-entry based on eligibility criteria. Four hundred and seventy-seven patients were ineligible with a total of 548 reasons stated; more than one reason for ineligibility could be present. The main reason for ineligibility was the size of the DCIS and/or margin involvement with 30% of all DCIS being considered too extensive for BCT, and 3% having involved margins. The percentage of patients with extensive DCIS or involved margins varied from 21 to 47% between the institutes (Table 2a).

One hundred and fifty-five of 433 eligible patients (36%) did not enter the trial because of doctors' or patients' preferences (Table 2b

**Table 2b:**
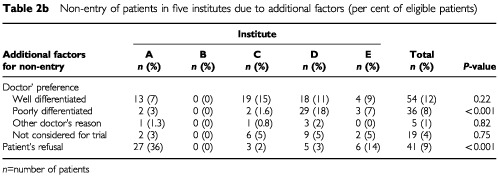
Non-entry of patients in five institutes due to additional factors (per cent of eligible patients

). In 26% of the eligible patients the doctor's preference for a particular treatment was the reason for non-entry (range 0–36%). Only in institute B no additional criteria for entry were applied: in the other institutes physicians often based a choice of therapy on the histologic differentiation type of DCIS. Forty-one (9%) of all eligible patients did not enter the trial because they refused to participate (range 0–36%, Table 2b).

Of all the 910 patients with DCIS 60% were treated with BCT (range 46–74% between the institutes). Fifty-one per cent of all the patients treated with BCT were entered in the trial. The treatment of the non-entered patients consisted of mastectomy in 57%, and BCT in 43% (Table 3

**Table 3:**
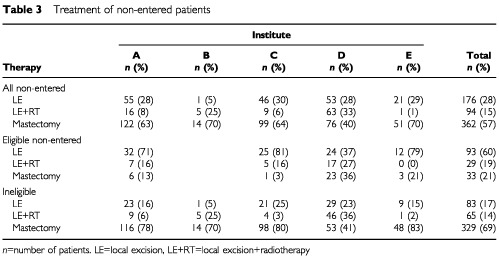
Treatment of non-entered patients

). As expected, most patients with DCIS that was considered too extensive were treated with mastectomy (95%). The proportion of patients who were treated conservatively outside the trial is similar in four institutes (30–36%), whereas in institute D 60% of the non-entered patients were treated with BCT. Here, many patients with an incomplete excision underwent radiotherapy instead of a re-excision or mastectomy (19 of 25 patients with involved margins treated with LE+RT). Eligible non-entered patients were generally treated with BCT (79%) without RT (60%). A notable exception again is institute D with only 64% treated conservatively, and 37% without RT (*P*<0.0001; Exact χ^2^ test in 3×4 table). In institute D mastectomy was the treatment of choice for ‘comedo-type’ DCIS (20 patients with ‘comedo-type’ DCIS were treated with mastectomy; nine with LE+RT). In institutes A and C, and to a lesser extent in institutes D and E, physicians preferred to treat well-differentiated DCIS cases outside the trial without radiotherapy (52 of 54 non-entered eligible patients were treated with LE only). In institute B, BCT usually consisted of LE+RT. Thirty-six of the 41 patients who refused to participate in the trial were treated with BCT.

Five hundred and sixty-five (63%) of the lesions were asymptomatic, detected by mammographic screening. The rate of mammographically detected DCIS varied from 47 to 77% between the institutes (58% in A, 75% in B, 56% in C, 77% in D and 47% in E). Twenty five per cent of the mammographically detected lesions were too large for BCT, compared with 40% of the clinically detected lesions (*P*<0.001). Consequently, patients who were eligible for the trial had a higher rate of mammographically detected lesions than the patients who were ineligible (74 *vs* 53%, *P*<0.001).

The median duration of follow-up for the trial patients was 51 months, for the non-entered patients 39 months. Follow-up was complete for 96% of the patients. The 4-year local recurrence-free interval rates are given in Table 4

**Table 4:**
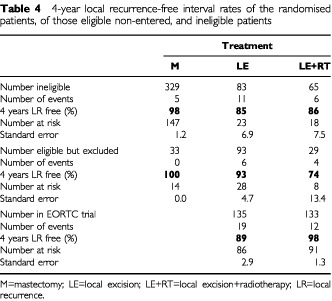
4-year local recurrence-free interval rates of the randomised patients, of those eligible non-entered, and ineligible patients

for the randomised patients, for the eligible non-entered, and ineligible cases. Five patients (1.4%) had a chest wall recurrence following mastectomy (*n*=362). At 4 years, the eligible non-entered patients treated with local excision alone had a 93% local recurrence-free interval, compared with 89% for the patients randomised to receive LE (s.e.=5.5, *P*=0.45). Four of the 29 eligible non-entered patients treated with LE+RT developed a local recurrence (4-year local recurrence-free interval 74%), compared to 12 of 133 of the LE+RT group in the trial (4-year local recurrence-free interval 98%) (s.e.=13.4, *P*=0.075) (Table 4). The majority of the recurrences in the LE+RT group in the trial occurred after 4 years.

When all non-entered patients – eligible and ineligible – are grouped together, those non-entered patients treated with LE+RT had a significantly worse 4-years local recurrence-free interval compared to those randomised for LE+RT (83 *vs* 98%, s.e.=6.9, *P*=0.031). The patients treated with LE alone had equal 4-years local recurrence-free intervals in and outside the trial (89%).

## DISCUSSION

The results of randomised clinical trials (RCTs) usually serve as the basis of treatment decisions, even though only a minority of the patient population will actually participate in trials (Fisher, 1991). Ellenberg (1994) has described a selection process of patients entered into RCTs together with the generalisation of results from randomised patients. From the population of patients with a disorder, only a subgroup may have the type of disease which is appropriate for the treatment under investigation. Of these, only part will be screened, only a proportion will be eligible for randomisation and some will not wish to enter the trial. The applicability of the trial results is highly dependent on the selection that occurs in each step.

Entry rates of patients for RCTs and the reasons for non-entry have been studied by a number of groups (Mccusker *et al*, 1982; Lee and Breaux, 1983; Martin *et al*, 1984; Anonymous, 1986; Jack *et al*, 1990; Fentiman *et al*, 1991; Gorst and Johnson, 1992; Kotwall *et al*, 1992; Kober and Torp-Pedersen, 1995; Partonen *et al*, 1996; Licht *et al*, 1997) but the effect of selection on the applicability of the trial results has been investigated in only a few, mainly pharmaceutical studies (Anonymous, 1986; Gorst and Johnson, 1992; Kober and Torp-Pedersen, 1995; Licht *et al*, 1997). To our knowledge, the effect of patient selection on applicability of results in surgical/radiotherapy trials has never been fully analysed. The present study showed that 52% of all patients with DCIS were not entered because they did not fulfil the eligibility criteria. The main reason for ineligibility was the extent of the DCIS, the most important criterion of suitability for BCT. Although 63% of the lesions was detected by mammography only, a considerable number (25%) of these lesions were still too large for BCT. As would be expected, a greater proportion (40%) of the clinically detected lesions was too large to be treated with BCT.

The variation between the institutes in the proportion of lesions treated with mastectomy may be caused by the differences in number of screen detected lesions. Also, institute D treated a number of cases with involved margins with BCT outside the trial, which may account for the lower proportion of lesions considered too large for BCT in this institute.

Other major reasons for ineligibility were a previous (breast) malignancy and the age of the patient. These eligibility criteria are necessary to ascertain complete follow-up of all patients, and to prevent the study from being contaminated by events unrelated to the DCIS. The non-entry of these patients is unlikely to have a major influence on the applicability of the trial results. The variation between the institutes in the number of patients ineligible due to a previous contralateral breast cancer may be caused by differences in follow-up policies for patients with breast cancer.

Additional criteria for selection were used in 36% of all eligible patients. Other studies report rates of eligible but non-entered patients of 25–73% (Lee and Breaux, 1983; Jack *et al*, 1990; Kotwall *et al*, 1992). The five institutes selected for our study are all specialised cancer centres and the physicians have put much effort into randomisation of suitable cases with only a few patients not being considered for entry. Hospitals with active oncology units have a higher accrual rate in cancer RCTs than other community hospitals (Mccusker *et al*, 1982). This may account for the lower proportion of eligible but non-entered patients compared with other studies. Three factors known to influence the entry of eligible patients in RCTs are problems with informed consent, and doctors' and patient's preference for one of the treatment arms in certain subgroups of the eligible patients (Taylor *et al*, 1984; Anonymous, 1986; Jack *et al*, 1990; Gorst and Johnson, 1992; Kober and Torp-Pedersen, 1995; Partonen *et al*, 1996; Licht *et al*, 1997) The reported rates of patient refusal vary from less than 10% to almost half of the eligible patients (Mccusker *et al*, 1982; Lee and Breaux, 1983; Martin *et al*, 1984; Jack *et al*, 1990; Kotwall *et al*, 1992). This variation is highly dependent on the type and stage of the disease, type of treatment, the patients' occupational level and the physicians' skills of informing the patient about the trial (Mccusker *et al*, 1982). In our study, large differences were seen in refusal rates between the various institutes, possibly reflecting differences in consent procedure. Williams and Zwitter (1994) reported large differences in informed consent used between Mediterranean and non-Mediterranean participating clinicians in European multicentre randomised clinical trials, with the latter being more likely to carry out full consent procedures than those from Mediterranean countries.

Personal perceptions on ‘optimal treatment policies’ may result in physicians' reluctance to randomise certain subgroups of patients for one of the treatment arms (Taylor *et al*, 1984). Our study shows that in four of the five institutes clinicians did not enter eligible patients based on the histologic subtype of DCIS. Results of non-randomised studies based on (morphologic) classification of DCIS (Page *et al*, 1982; Lagios *et al*, 1989; Silverstein *et al*, 1990; Bellamy *et al*, 1993; Eusebi *et al*, 1994) have influenced the participating clinicians' opinions on the treatment of subtypes of the disease. Of all randomised patients the histology was reviewed. The preference of institute D to treat comedo-type DCIS with mastectomy or radiotherapy outside the trial resulted in the observation that of all randomised patients from institute D 78% had well differentiated DCIS (according to a classification based on cytonuclear factors (Holland *et al*, 1994)); in other institutes the rate of the three subtypes is more equally distributed. Consequently, the randomised patients may not be representative for all eligible patients with respect to differentiation type. The risk factor analysis of the EORTC 10853 trial showed that radiotherapy reduced the risk of recurrence in all histological types of DCIS (Bijker *et al*, 2001).

A randomised trial comparing mastectomy with BCT for DCIS has never been conducted and it is unlikely that this will ever occur. Nevertheless, from all non-randomised studies there is strong evidence that mastectomy has significantly lower local recurrence rates as compared to any form of BCT. This study shows that for optimal local control, still a substantial number of patients (40%) were offered a mastectomy, which is a reasonable option as this large series shows only a 2% chest wall recurrence rate at 4 years. This is consistent with the reported rates, ranging from 1 to 4% (Cataliotti *et al*, 1992; Silverstein *et al*, 1995; Warneke *et al*, 1995).

When the outcome of BCT of non-entered patients is compared with that of the randomised patients, there seems to be a larger beneficial effect of RT on local control for those treated in the trial. Although the short duration of follow-up does not permit us to draw any definite conclusions from this finding, it suggests that patient selection has caused this effect. Many patients with a presumed worse prognosis, like those with involved margins, were treated with LE+RT outside the trial. This may explain why the 4-year local recurrence rates in the non-entered patients treated with LE+RT are relatively high compared to the patients in the trial treated with LE+RT. It might also indicate the importance of a complete excision, even with the application of RT (Silverstein *et al*, 1999; Bijker *et al*, 2001). In contrast, the non-entered patients treated with local excision alone had more similar 4-years local recurrence free rates in comparison to those patients treated with local excision in the trial, suggesting that LE alone is employed in those non-entered patients who were considered to have a low risk for local recurrence.

A few other studies have shown that the outcome of patients treated within RCTs is better compared to those undergoing the same treatment outside trials, which is mainly explained by ‘positive’ selection of patients: patients with a presumed worse prognosis are more likely to be excluded from participation (Anonymous, 1986; Gorst and Johnson, 1992: Kober and Torp-Pedersen, 1995; Licht *et al*, 1997). The only cancer RCTs that have shown this effect are two leukaemia trials; of two other studies that were found one investigated the effect of selection on outcome of antidepressant treatment and one on a pharmaceutical intervention after acute myocardial infarction.

Two important questions can be addressed related to the generalisability of the results of this trial. (1) For what proportion of patients with DCIS BCT is a reasonable option with a low risk of recurrence, and for whom a mastectomy is preferred because of the extent of the DCIS? (2) Do all patients with DCIS benefit from RT after local excision for DCIS? We were able to study patient selection in five institutes. Our study shows that of all patients with DCIS, only 70% could be offered BCT. Although trial participation was considered in almost all eligible patients, only two thirds were entered, with large differences between the centres and varying reasons for non-entry. Randomised patients treated with LE+RT had better 4-year local recurrence-free rates than those treated with LE+RT outside the trial. This indicates that the quantitative results of this trial (i.e. the reduction of the 4-years local recurrence rate with 38% by the application of RT) might not be generalisable to all patients with DCIS eligible for BCT. Especially disconcerting is the observed selection with respect to the differentiation type. Our findings indicate the importance of prospective registration and follow-up of non-randomised patients for evaluating the applicability of the trial results to the general population with the disease (Ellenberg, 1994; Olschewski and Scheuren, 1985). Before committing an institute to participation, future trials might further need a quality assessment of the candidate centre including a review of a possible predetermined position regarding the treatment to be investigated in the study.
